# Acute Viral Myocarditis in a Young Adult: A Case Report

**DOI:** 10.7759/cureus.66325

**Published:** 2024-08-06

**Authors:** Jason Nguyen, Cristina Benites, Daniel Karpel, Mohammed Akram, Livasky Concepcion

**Affiliations:** 1 Internal Medicine, HCA Florida Aventura Hospital, Aventura, USA; 2 Department of Medical Education, Nova Southeastern University Dr. Kiran C. Patel College of Allopathic Medicine, Davie, USA

**Keywords:** myocarditis, hapatopathy, cardiomyopathy, coxsackievirus, adenovirus, gastroenteritis, chocardiology, heart failure, viral etiology, young adults

## Abstract

Viral myocarditis is a serious complication of viral infections that is known to impact young adults and can result in significant cardiac issues like earlier onset of heart failure, arrhythmia, or structural heart disease if not detected and treated promptly. This is a case report focusing on a 25-year-old woman diagnosed with viral myocarditis highlighting the diagnostic difficulties it can present with due to its diverse symptoms. Following a thorough diagnostic workup and appropriate treatment, including diuretics, antibiotics, and guideline-directed medical therapy, her condition significantly improved. Early suspicion and prompt treatment are important because myocarditis can lead to long-term heart failure. This case shows the nature of the symptoms and the challenges posed by current diagnostic methods. By conducting clinical assessments and using advanced imaging techniques, the diagnosis was confirmed, leading to appropriate treatment for this patient.

## Introduction

Myocarditis is an inflammatory disorder that is known to affect young adults and can have severe consequences like heart failure, arrhythmia, or structural heart disease if left untreated [1-3]. It is frequently seen as a consequence of bacterial, viral, or parasitic infection and is an established primary cause of dilated cardiomyopathy [3,4]. Despite being a well-known contributor to congestive heart failure, there is a lack of conventional techniques available in the acute setting to aid in its diagnosis. This makes diagnosing acute viral myocarditis a complex process [5]. Finding effective treatments has proven to be a challenge due to the complex nature of chronic dilated cardiomyopathy following viral myocarditis, which is influenced by factors like the patient's genetic makeup, viral genetics, and environmental conditions [6]. We discuss the case of a 25-year-old woman presenting with acute viral myocarditis complicated with gastroenteritis and congestive hepatopathy. This case report explores one of the different ways in which viral myocarditis caused by Coxsackie B can manifest itself and the importance of early clinical suspicion, diagnosis, and management. 

## Case presentation

A 25-year-old Hispanic female with no significant past medical or family history or heart disease presented to the emergency department for new onset shortness of breath and one week of progressively worsening abdominal bloating and watery diarrhea. She lives an active lifestyle, regularly exercising without experiencing any pains or dyspnea on exertion. The last known travel was from South America to the United States over one year ago. She had no sick contacts at home and owned no pets. Physical exam was significant for decreased breath sounds in the lung bases bilaterally, a holosystolic murmur at the left sternal border, and 1+ pitting edema of the upper and lower extremities.

**Figure 1:**
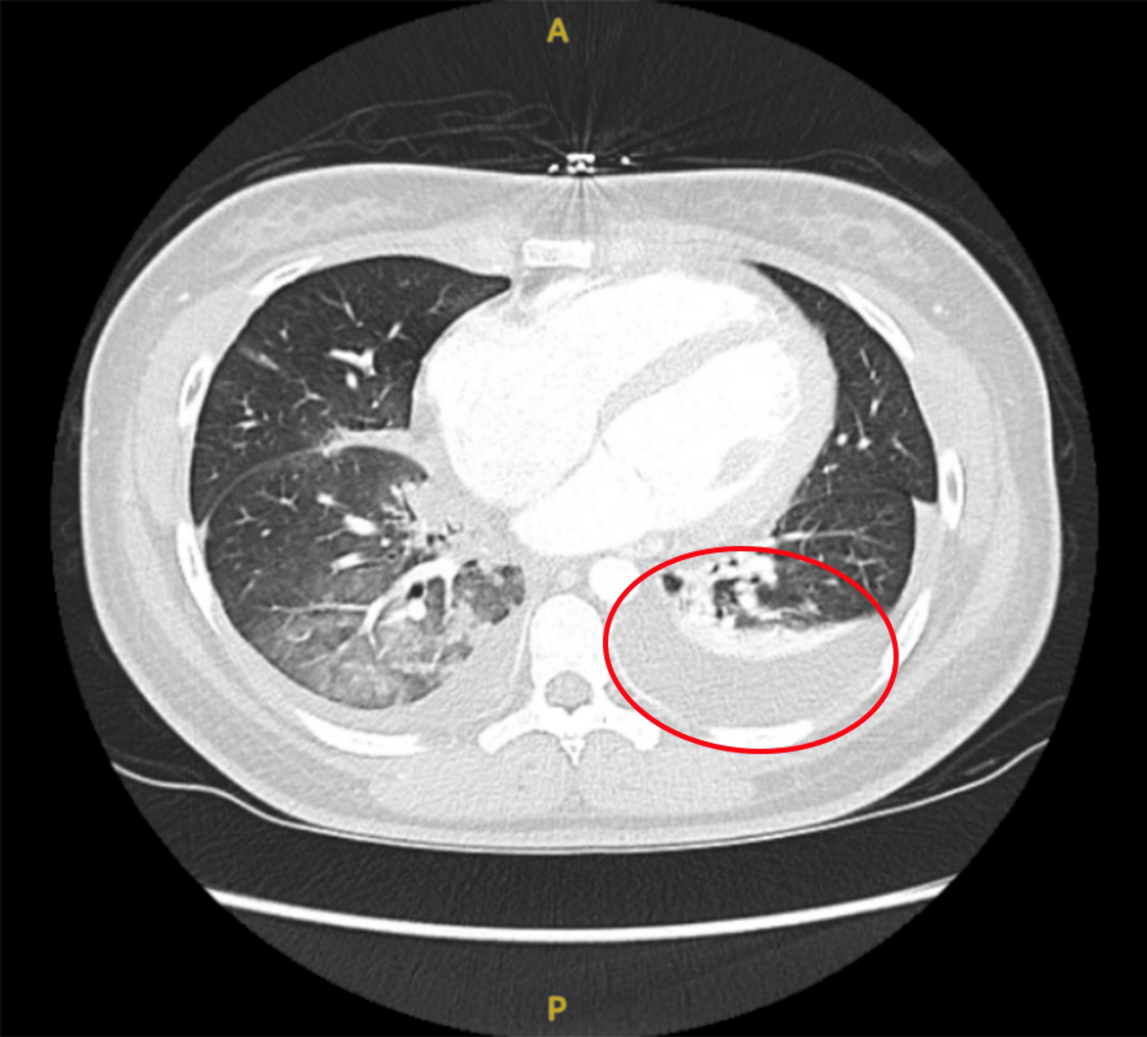
CT scan (transverse section) CT scan of the chest taken after diuresis and right thoracentesis shows bilateral pleural effusions, with fluid in both pleural cavities, more prominent on the right. The posterior right lower lobe (RLL) has consolidation (red circle), indicating lung tissue filled with liquid. There is also bilateral compressive atelectasis in the posterior segments, where lung tissue is collapsed due to the pressure from the effusions.

**Figure 2:**
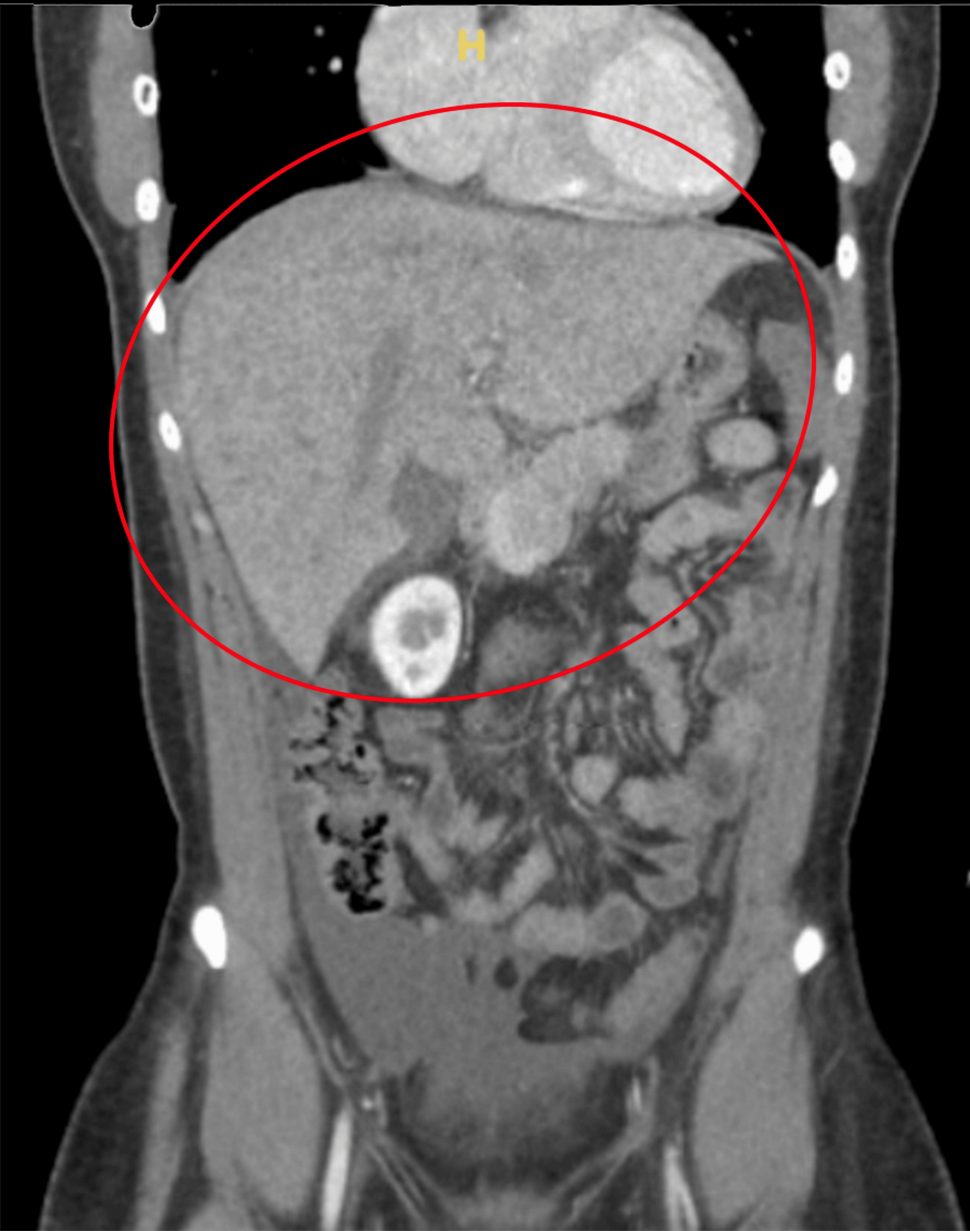
CT scan (coronal section) CT scan of the abdomen and pelvis (CTAP) showing hepatomegaly extending beyond its normal boundaries. There is also a noticeable presence of ascites, with fluid accumulation visible in the peritoneal cavity, surrounding the liver and intestines.

**Figure 3:**
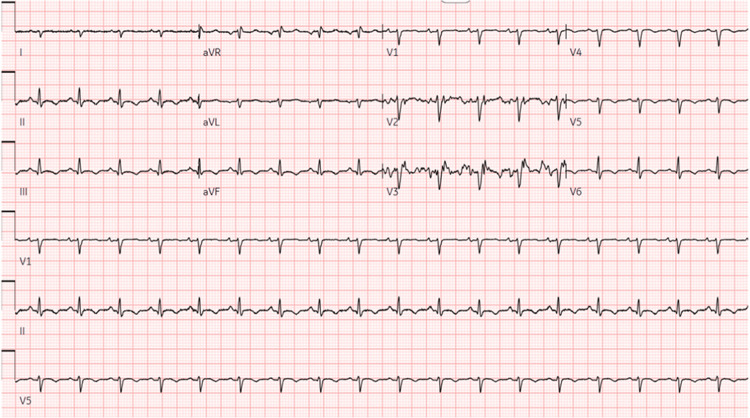
ECG of patient ECG shows right-axis deviation, nonspecific intraventricular block, and nonspecific T-wave abnormalities.

**Figure 4:**
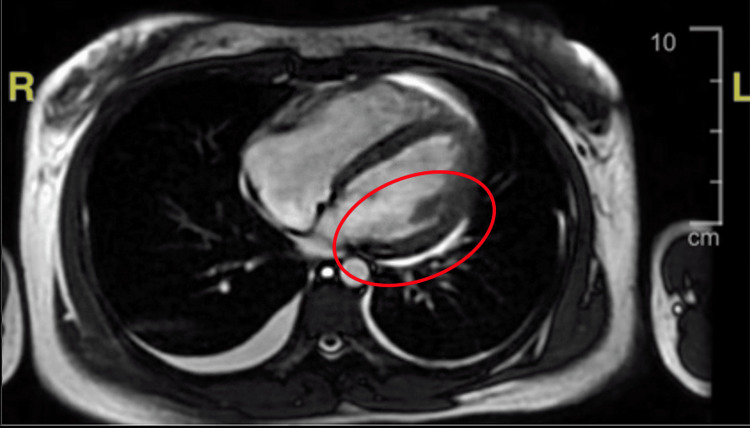
Cardiac MRI (transverse section) Inflammation can be seen as white patches (red circle) within the myocardium after contrast injection. Eccentric dilation of ventricles can be appreciated

The initial set of labs revealed severely elevated pro-brain natriuretic peptide (BNP), elevated liver enzymes, low albumin, prolonged prothrombin time (PT) and international normalized ratio (INR), elevated C-reactive protein (CRP), and elevated lactate dehydrogenase (LDH). Pleural fluid analysis revealed normal fluid protein and elevated fluid LDH. Chest X-ray showed bilateral pleural effusions, with right-sided dominance. Abdomen and chest computed tomography (CT) (Figures 1, 2) showed right middle and lower lobe pneumonia, hepatomegaly which was suggestive of hepatitis, and small-volume ascites. Electrocardiogram (ECG) (Figure 3) had right-axis deviation, nonspecific intraventricular block, and nonspecific T-wave abnormalities. No prior ECG tracings were available to compare. Transthoracic echocardiogram (TTE) with Doppler significant for an estimated ejection fraction 20% to 25% and mildly elevated end-diastolic internal diameters of the left and right ventricles. Cardiac catheterization showed no coronary artery disease, so she was subsequently diagnosed with non-ischemic dilated cardiomyopathy. Cardiac magnetic resonance (CMR) (Figure 4) was suggestive of acute inflammatory myocarditis. Respiratory viral titers were reactive for adenovirus, Coxsackie type B4, Coxsackie type B5, and Coxsackie type B6 (Table 1).

**Table 1:** Lab values The table shows the laboratory results of the patient. Abnormalities include a markedly elevated pro-brain natriuretic peptide (BNP) at 7,270 pg/mL, elevated liver enzymes (aspartate aminotransferase (AST) at 792 U/L and alanine transaminase (ALT) at 878 U/L), low albumin at 2.9 g/dL, prolonged prothrombin time (PT) at 14.3 seconds, slightly elevated international normalized ratio (INR) at 1.3, elevated C-reactive protein (CRP) at 2.9 mg/dL, elevated lactate dehydrogenase (LDH) at 410 U/L, and elevated lactic acid at 2.4 mmol/L. Additionally, the presence of adenovirus and Coxsackie B4, B5, and B6 antibodies are noted, all at a titer of 1:8 or higher, which exceeds the negative threshold.

Parameter	Obtained Value	Reference Range
Pro-brain natriuretic peptide (BNP)	7,270 pg/mL	0-217 pg/mL
AST	792 U/L	10-40 U/L
ALT	876 U/L	10-60 U/L
Albumin	2.9 d/dL	3.5-5.0 g/dL
Prothrombin time (PT)	14.3 seconds	11-13.5 seconds
INR	1.3	0.8-1.1
C-reactive protein (CRP)	2.9 mg/dL	0-1.0 mg/dL
Lactate dehydrogenase (LDH)	410 U/L	84-246 U/L
Lactic acid	2.4 mmol/L	0.4-2.0 mmol/L
Adenovirus antibody	1:8	< 1:8
Coxsackie Type B4 antibody	1:8	Negative - < 1:8
Coxsackie Type B5 antibody	1:16	Negative - < 1:8
Coxsackie Type B6 antibody	1:16	Negative - < 1:8

With the presence of exudative pleural effusions and ascites, the patient received loop diuretics with improvement of her presenting symptoms. Despite this, the pleural effusions persisted, so thoracentesis was performed with subsequent resolution of the shortness of breath. She was given azithromycin and ceftriaxone for concurrent pneumonia, and subsequently switched to oral doxycycline. Once medically stable, she was initiated on metoprolol succinate. Due to hypotension, an angiotensin-converting enzyme (ACE) inhibitor and a mineralocorticoid receptor antagonist (MRA) were not started in the hospital. After being discharged from the hospital, the patient followed up closely with her cardiologist and was started on a valsartan-sacubitril combination pill in addition to the metoprolol succinate. Her fatigue and lower extremity edema had greatly improved since she was at the hospital with a reported six-month follow-up TTE showing improved ejection fraction.

## Discussion

In the spectrum of inflammatory cardiomyopathies, infectious myocarditis is a predominant subtype, especially in developing countries where there is a broad spectrum of parasitic and bacterial etiologies [1]. Early signs of myocarditis may include symptoms such as dyspnea, chest pain, fatigue, and, in the majority of cases, a recent history of upper respiratory symptoms. These presenting symptoms can make diagnosis difficult due to the myriad of pathologies that can present with these same symptoms. The incidence of myocarditis is estimated to be about 6.1 cases per 100,000 men and 4.4 per 100,000 in women aged 35 to 39 years [2]. Its diagnosis is often missed due to varied symptoms and a lack of noninvasive tests. Acute myocarditis usually has a good short-term prognosis, but long-term risks include earlier onset of heart failure.

Diagnosis relies on cardiac MRI and endomyocardial biopsy (EMB). CMR is useful in identifying local sites of inflammation within cardiac tissue; however, it does not offer specific etiologies causing the inflammation [7]. Despite EMB being the gold standard in diagnosis, performing it has risks, as with any invasive procedure. For this reason, EMB is recommended to be utilized for patients with critical presentations, such as cardiogenic shock, ventricular arrhythmias, or high-degree heart blocks, or if there is a high suspicion of involvement from immunosuppressive agents that would require further treatment optimization, none of which was present in our patient [7]. Even with these indications, EMB is a class C recommendation and should be further investigated to determine the broadened utility of the procedure [7]. 

The significance of taking viral infections into account as a potential cause when dealing with hepatitis and myocarditis in a patient is one important aspect that should not be overlooked. Although Coxsackievirus is the most likely cause of myocarditis, we could not rule out the involvement of adenovirus. Studies have found that the Coxsackie B-adenovirus receptor (CAR) is responsible for viral infection in cardiac tissue for both Coxsackie B and adenovirus [8,9]. Coxsackievirus B's diverse effects highlight the need for a careful diagnosis.

Viral infections can also cause gastroenteritis, and it is important also to consider it as part of other differential diagnoses. While these are important, the primary focus in management was optimizing cardiomyopathy treatment. This approach aims to prevent worsening right heart failure and further deterioration of liver function and avert the risk of cirrhosis [10,11]. Therefore, managing the cardiac aspect effectively is crucial to prevent secondary hepatic complications.

## Conclusions

Acute myocarditis is a relatively uncommon complication secondary to an infectious process and primarily affects young adults, making diagnosis difficult. Diagnostic modalities like CMR and endomyocardial biopsy are useful in assessing cardiovascular risk and can help guide long-term treatment; however, they are infrequently utilized in the acute setting due to their narrow spectrum of use and the invasive nature of the procedure, respectively. Although typically favorable in short-term prognosis, there is a delayed risk of early-onset congestive heart failure, which can lead to morbidities such as pulmonary hypertension, portal hypertension, and subsequently cirrhosis. For young patients with signs of CHF, such as the one seen in this case report, the possibility of viral myocarditis should be highly considered. Maintaining early clinical suspicion, even in cases of generally favorable presentation, can be essential to appropriately diagnose and treat the condition. Close outpatient follow-up is important to minimize the long-term effects of myocarditis.
